# Trends and Factors Associated with Medication-Related Patient Safety Incidents Reported in Korean Hospitals: A Retrospective National Analysis (2020–2024)

**DOI:** 10.3390/healthcare14070927

**Published:** 2026-04-02

**Authors:** Jieun Shin, Wanjin Hwang, Nam-Yi Kim

**Affiliations:** 1 Department of Biomedical Informatics, College of Medicine, Konyang University, Daejeon 35365, Republic of Korea; jeshin@konyang.ac.kr; 2 Department of Thoracic and Cardiovascular Surgery, Konyang University Hospital, Daejeon 35365, Republic of Korea; 200687@kyuh.ac.kr; 3 Department of Nursing, Konyang University, Daejeon 35365, Republic of Korea

**Keywords:** medication errors, patient safety, adverse drug events, incident reporting systems, hospitals, risk factors

## Abstract

**Background:** Medication-related patient safety incidents are among the most common and preventable sources of harm in healthcare systems worldwide. In Korea, medication incidents have consistently ranked among the most frequently reported patient safety events since the implementation of the national patient safety reporting system. However, national-level evidence examining recent trends and factors associated with clinically significant harm remains limited. **Methods**: This retrospective observational study analyzed medication-related patient safety incidents reported to the Korea Patient Safety Reporting & Learning System (KOPS) from 2020 to 2024. KOPS operates as a hybrid system with voluntary reporting for general incidents and mandatory reporting for severe events since 2021. Harm severity was dichotomized into near-miss incidents and adverse/sentinel events. Of 36,281 reported incidents, 9495 were included after excluding cases with missing key variables. This dichotomization was applied to distinguish clinically meaningful harm and support robust statistical analysis. **Results**: Medication-related incidents showed an increasing trend in reported cases over time (annual percent change: 15.38%; 95% CI, 6.0–25.6%). Among the analyzed cases, 21.2% resulted in adverse/sentinel events. These events were more frequently observed in large hospitals, emergency and critical care settings, and during evening and nighttime periods. This increase may reflect changes in reporting practices following mandatory reporting policies, as well as potential changes in medication-related risks. **Conclusions**: Reported medication-related incidents are increasing, and a substantial proportion are associated with harm. However, these findings should be interpreted cautiously, as they reflect reported incidents rather than the true incidence of medication errors. Targeted strategies focusing on high-risk settings (emergency/critical care), vulnerable patient groups, and off-hour periods may be needed. Integrating risk-based approaches into national patient safety policies may help reduce preventable harm.

## 1. Introduction

Medication-related patient safety incidents, including medication errors and adverse drug events (ADEs), are among the most common and preventable causes of harm in healthcare systems worldwide [1,2,3]. Medication errors refer to preventable failures in the medication use process (e.g., prescribing, dispensing, or administration), whereas adverse drug events (ADEs) refer to injuries resulting from medication use, which may or may not be preventable. This conceptual distinction is important because not all medication errors result in harm, and not all ADEs are caused by errors. In response to this burden, the World Health Organization (WHO) launched the Medication Without Harm Global Patient Safety Challenge in 2017, identifying medication-related harm as a major source of avoidable patient injury and estimating the associated global cost at approximately USD 42 billion annually [1]. International evidence indicates that approximately one in ten patients experience preventable harm across healthcare settings, with around 12% of these events classified as severe, and medication-related incidents accounting for nearly one quarter of all preventable harm [2,3]. In England alone, an estimated 237 million medication errors occur annually across the medication-use process, of which approximately 66 million are considered potentially clinically significant, contributing to substantial healthcare costs and over 1700 associated deaths each year [4]. These findings underscore medication safety as a core component of healthcare quality and patient safety improvement.

National incident reporting and learning systems are widely used to monitor medication-related safety risks and support evidence-based prevention strategies [5,6]. However, incident reporting systems inherently do not capture all medication errors, as reporting is often voluntary and influenced by institutional reporting culture and practices, which may lead to underreporting. Despite these limitations, analyses of large reporting databases have demonstrated their value in identifying temporal trends, high-risk patient groups, and clinical settings associated with increased harm severity [6,7,8]. Previous research has consistently shown that patient age, clinical department, institutional complexity, and care environment—particularly emergency and critical care settings—are strongly associated with medication-related incident severity [7,8,9]. However, the generalizability of these findings to the Korean healthcare system may be limited due to differences in healthcare delivery structure, reporting systems, and patient safety culture. In particular, inter-institutional variability in reported incidents may reflect both structural differences in care complexity and variations in reporting practices across institutions.

In Korea, medication-related incidents have remained among the most frequently reported patient safety events since the enactment of the Patient Safety Act in 2016 and the establishment of the Korea Patient Safety Reporting & Learning System (KOPS) [10]. Medication incidents and patient falls have consistently ranked as the top two reported categories, highlighting persistent vulnerabilities in routine medication use processes [11]. KOPS introduced mandatory reporting for serious incidents resulting in severe harm or death in January 2021, while maintaining voluntary reporting for less severe events [10]. Despite improved nationwide data availability, underreporting and inter-institutional variability remain inherent limitations [5,12]. This study adopts an exploratory analytical approach to examine nationwide trends and associations, rather than testing a single predefined hypothesis. The primary outcome of interest is harm severity (near-miss vs. adverse/sentinel events), while secondary analyses explore variations across patient, institutional, and incident characteristics.

Policy-level attention to medication safety in Korea has been strengthened through the Second Comprehensive Patient Safety Plan (2023–2027), which identifies medication errors as a national priority and emphasizes strengthening medication management systems, patient safety education, reporting mechanisms, and digital safety technologies [11]. These directions align with the WHO Global Patient Safety Action Plan 2021–2030 [13].

Despite these efforts, empirical evidence on medication-related patient safety incidents in Korea remains limited. Existing studies have largely focused on single institutions, specific medication classes, or short observation periods, limiting generalizability [14,15]. For example, a national study using KOPS data (2017–2019) primarily examined overall trends and general characteristics of patient safety incidents, without focusing specifically on medication-related incidents or harm severity across multiple contextual factors [16]. In addition, studies conducted in long-term care hospitals or specific clinical settings have explored factors associated with patient safety incidents but were limited to specific populations or institutional contexts and did not incorporate comprehensive multi-level variables [17]. These previous studies have provided important descriptive insights; however, they have generally not simultaneously examined patient-level (e.g., age, sex), institutional-level (e.g., hospital type, bed capacity), and incident-level (e.g., time of occurrence, clinical department) factors in relation to harm severity using multi-year nationwide data. Furthermore, few studies have explicitly distinguished clinically meaningful harm (adverse/sentinel events) from near-miss incidents to identify determinants of harm severity.

Although some nationwide analyses exist, few have examined harm severity in relation to patient demographics, clinical departments, and institutional characteristics over multiple years. In addition, previous studies have not comprehensively integrated patient-, institutional-, and incident-level factors to identify determinants of clinically meaningful harm using multi-year national data. While international studies have identified these factors as key determinants of medication-related harm severity [7,8,9], multi-year national analyses within the Korean context remain scarce.

Therefore, the present study extends previous national analyses by (1) focusing specifically on medication-related incidents; (2) integrating patient-, institutional-, and incident-level variables within a unified analytical framework; and (3) examining harm severity using five years of nationwide data (2020–2024), including the period following the introduction of mandatory reporting for severe incidents. By doing so, this study provides more comprehensive and context-specific evidence on the determinants of clinically meaningful harm in the Korean healthcare system.

To address these gaps, this study analyzes five years (2020–2024) of nationwide medication-related patient safety incident reports from KOPS. The objectives are to describe national trends, examine harm severity by patient and clinical characteristics, identify factors associated with adverse/sentinel events, and explore implications for medication safety management and national patient safety policy. By explicitly addressing these gaps, this study provides new evidence on the patterns and determinants of harm severity using multi-year national reporting data. By integrating multi-year national data, this study aims to provide context-specific evidence to inform risk-based medication safety strategies in the Korean healthcare system.

## 2. Methods

### 2.1. Study Design and Data Source

This study employed a retrospective observational design using national patient safety incident data reported to the Korea Patient Safety Reporting & Learning System (KOPS) between 1 January 2020 and 31 December 2024. KOPS is a nationwide incident reporting system established under the Korean Patient Safety Act and operated by the Ministry of Health and Welfare. The system collects standardized reports of patient safety incidents from healthcare institutions across Korea, including information on patient characteristics, healthcare settings, incident type, and harm severity. Reports are submitted through a web-based platform using a standardized reporting format that captures structured information on incident characteristics and outcomes. The system covers a wide range of healthcare institutions, including clinics, hospitals, general hospitals, and tertiary hospitals. Incident severity is classified according to predefined categories, including no harm, near miss, mild harm, moderate harm, severe harm, and death.

During the study period, KOPS operated as a hybrid reporting system, consisting of voluntary reporting for general patient safety incidents and mandatory reporting for serious incidents resulting in severe harm or death, which was introduced in January 2021. KOPS data are subject to standardized reporting formats and undergo basic data quality checks at the institutional and national levels; however, the dataset does not include denominator information such as total admissions or patient-days, limiting the ability to estimate incidence rates.

### 2.2. Study Population and Case Selection

Among all patient safety incidents reported to KOPS from 2020 to 2024, a total of 36,281 medication-related incidents were initially identified. Medication-related incidents were operationally defined as events categorized under medication-related incident types in the KOPS classification system, including errors occurring during prescribing, dispensing, preparation, or administration processes. Incidents reported from healthcare institutions with clearly identifiable types—tertiary hospitals, general hospitals, hospitals, and clinics—were retained, yielding 28,201 cases for further screening.

**Figure 1:**
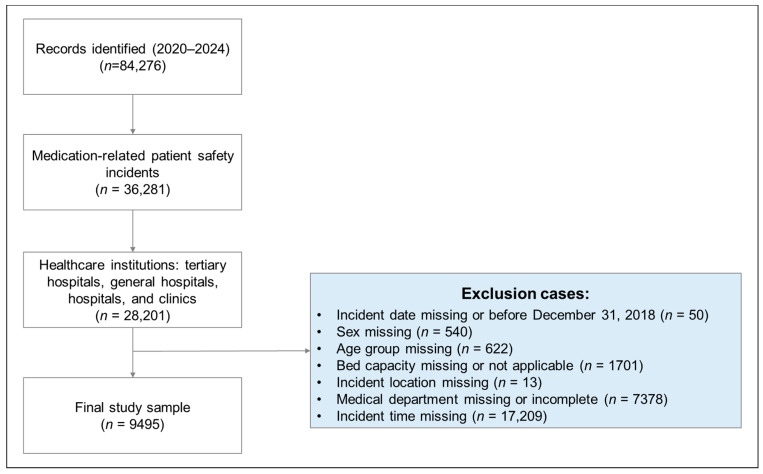
Flow diagram of study sample selection from the Korea Patient Safety Reporting & Learning System (KOPS), 2020–2024.

Cases were excluded if they met any of the following criteria: unclear incident date or occurrence before 31 December 2018 (*n* = 50); missing sex information (*n* = 540); missing age group (*n* = 622); missing or unclassifiable hospital bed size (*n* = 1701); missing incident location (*n* = 13); missing or incomplete clinical department information (*n* = 7378); or missing incident time (*n* = 17,209). After applying these exclusion criteria, 9495 medication-related incidents were included in the final analysis. The case selection process is summarized in Figure 1.

The exclusion of cases with missing incident time (*n* = 17,209) was necessary because the time of occurrence was a key independent variable in the analysis. However, this substantial exclusion may introduce selection bias, as reporting completeness may differ across institutions or clinical settings. In addition, the excluded records may differ systematically from the final analytic sample. For example, cases with missing incident time or clinical department information may be more likely to originate from specific healthcare settings or reflect variations in institutional reporting practices, which could influence the distribution and severity of reported incidents. Because the present study relied on secondary national reporting data with substantial missingness in key analytic variables, complete-case analysis was considered the most transparent and interpretable approach for the primary model, although this may have reduced the representativeness of the final analytic sample.

### 2.3. Measures and Variables

#### 2.3.1. Outcome Variable

The outcome variable was the level of harm associated with medication-related patient safety incidents. In the original KOPS classification system, harm severity is categorized into six levels: no harm, near miss, mild harm, moderate harm, severe harm, and death. For the purposes of this study, harm severity was recategorized to support robust statistical analysis and alignment with commonly used patient safety research frameworks.

In the primary analysis, harm severity was dichotomized into near-miss and adverse/sentinel events. Although harm severity is inherently ordinal, dichotomization was applied to distinguish clinically meaningful harm from non-harm events and to ensure sufficient statistical power and model stability, given the relatively small number of severe and sentinel events. Near-misses included incidents classified as ‘no harm’ or ‘near miss’, referring to events that did not reach the patient or reached the patient without causing harm. Adverse/sentinel events included incidents classified as mild, moderate, severe harm, or death, representing events that resulted in patient harm ranging from temporary injury requiring clinical intervention to serious harm or death.

Sentinel events (severe harm or death) accounted for a small proportion of cases; therefore, they were combined with adverse events to avoid sparse data bias in regression analysis. While this approach improves statistical power and model stability, it may reduce the granularity of clinical severity by combining events with different levels of harm. However, this approach is consistent with prior patient safety research that distinguishes clinically meaningful harm from non-harm events. This analytic strategy was intended to balance clinical interpretability with statistical robustness in the context of sparse severe-event categories within the national reporting dataset. All recoding procedures were applied consistently across reporting years and were documented to ensure reproducibility.

#### 2.3.2. Independent Variables

The original KOPS dataset contains a broad range of variables, including diagnosis, corrective actions following the incident, reporter information, and date of discovery. Based on previous national and international studies examining patient safety incidents and severity [16], as well as guidelines from patient safety agencies emphasizing key contextual risk factors [17], variables with potential relevance to the characteristics and severity of medication-related patient safety incidents were selected for analysis. In addition, variable selection was informed by the Systems Engineering Initiative for Patient Safety (SEIPS) framework, which emphasizes the interaction between patient, task, environment, and organizational factors in influencing patient safety outcomes [18].

The independent variables included patient-, institutional-, and incident-related characteristics. Patient characteristics comprised sex (male, female) and age group (0–19, 20–59, and ≥60 years). Institutional characteristics included the type of healthcare institution (clinic, hospital, general hospital, or tertiary hospital) and hospital bed capacity (<200, 200–499, ≥500 beds, or no inpatient beds). Hospital bed capacity was categorized based on commonly used thresholds in the Korean healthcare system classification and prior patient safety research, reflecting differences in institutional size and care complexity. Clinical context was represented by the medical department, categorized as medical disciplines, surgical disciplines, diagnostic and support services, emergency and critical care, pediatrics, psychiatry and mental health, and others. These categories were defined by grouping related clinical specialties according to functional roles and care processes within healthcare systems, with emergency and critical care separated due to their high-acuity and time-sensitive nature. Incident-related characteristics included time of occurrence, categorized as daytime (07:00–14:59), evening (15:00–22:59), and nighttime (23:00–06:59).

### 2.4. Statistical Analysis

Descriptive statistics were used to summarize the characteristics of medication-related patient safety incidents, and frequencies and percentages were calculated for categorical variables. To examine temporal trends in the proportion of medication-related incidents over the five-year period, a log-linear regression model assuming a Poisson distribution was applied to estimate the annual percent change (APC) with 95% confidence intervals (CIs).

Differences in harm severity (near-miss vs. adverse/sentinel events) according to patient, institutional, and incident characteristics were assessed using the chi-square (χ^2^) test. To identify factors independently associated with adverse/sentinel events, multiple logistic regression analysis was performed. Harm severity was entered as the dependent variable, while sex, age group, type of healthcare institution, hospital bed size, medical department, and incident time were included as independent variables. Prior to model estimation, multicollinearity among independent variables, particularly between the type of healthcare institution and hospital bed size, was assessed using variance inflation factors (VIFs), and no significant multicollinearity was identified (VIF < 5). Results are presented as odds ratios (ORs) with 95% CIs. All statistical analyses were conducted using R software (version 4.4.0) and SPSS Statistics (version 26.0; IBM Corp., Armonk, NY, USA), with a significance level set at *p* < 0.05. Both R and SPSS were used to ensure analytical reproducibility and cross-validation of results across statistical platforms. Model fit was evaluated using standard goodness-of-fit measures, including the Hosmer–Lemeshow test. In addition, key assumptions of logistic regression, including linearity in the logit for continuous variables and the absence of influential outliers, were examined to ensure the robustness of the model. Because incident reports were collected from multiple healthcare institutions, observations may not be fully independent due to potential clustering at the institutional level. This was not explicitly modeled in the analysis and may influence the estimated associations.

## 3. Results

### 3.1. Trends in Medication-Related Patient Safety Incidents (2020–2024)

A total of 36,281 medication-related patient safety incidents were reported to the Korea Patient Safety Reporting & Learning System (KOPS) between 2020 and 2024. Over the study period, both the number and proportion of medication-related incidents increased steadily among all reported patient safety incidents. From 2022 onward, medication-related incidents comprised nearly half or more of all reported patient safety incidents nationwide. Detailed annual numbers and proportions are presented in Table 1.

Trend analysis using a log-linear regression model demonstrated a significant upward trend in the proportion of medication-related incidents over the five-year period. The APC was estimated using a log-linear Poisson regression model with calendar year as a continuous variable and the proportion of medication-related incidents as the outcome. The annual percent change (APC) was 15.38% (95% confidence interval [CI], 6.0–25.6%). This increasing trend should be interpreted with caution, as it may reflect both improvements in reporting practices following the introduction of mandatory reporting for severe incidents in 2021 and a potential increase in medication-related risks.

### 3.2. Harm Severity of Medication-Related Incidents According to Patient, Institutional, and Incident Characteristics

Among the 9495 medication-related patient safety incidents included in the final analysis, 7484 cases (78.8%) were classified as near-miss incidents, while 2011 cases (21.2%) were classified as adverse/sentinel events. Sentinel events (severe harm or death) accounted for a small proportion of cases and were therefore analyzed in combination with adverse events. The distribution of harm severity varied significantly across the study period (χ^2^ = 282.382, *p* < 0.001). The proportion of near-miss incidents accounted for the majority of cases in all years, whereas the proportion of adverse/sentinel events ranged from 9.4% in 2020 to a peak of 26.1% in 2023, before decreasing to 15.3% in 2024. Differences in harm severity were observed according to patient characteristics. Although no significant difference was found by gender (χ^2^ = 0.489, *p* = 0.485), harm severity differed significantly by age group (χ^2^ = 33.818, *p* < 0.001). The proportion of adverse/sentinel events was highest among patients aged ≥ 60 years (23.0%), whereas near-miss incidents were most frequent in the 0–19 years age group (84.3%). However, in multivariable analysis, younger age groups showed higher odds of adverse/sentinel events compared with the reference group, suggesting that crude proportions and adjusted associations differ due to confounding factors. More broadly, discrepancies between descriptive (unadjusted) findings and multivariable (adjusted) results may arise because the distribution of patient, institutional, and clinical characteristics differs across comparison groups. Therefore, adjusted estimates should be interpreted as reflecting independent associations after controlling for these factors, whereas descriptive results represent unadjusted distributions and may be influenced by confounding. This discrepancy may be explained by differences in the distribution of institutional and clinical characteristics across age groups. For example, older patients are more likely to receive care in larger hospitals or high-acuity settings, which are themselves associated with higher rates of adverse events. After adjusting for these factors in the regression model, the independent effect of age may appear reduced or reversed. Therefore, the results of the multivariable analysis should be interpreted as reflecting independent associations after adjustment for confounding factors, whereas descriptive findings represent unadjusted distributions and should be interpreted with caution.

Significant differences in harm severity were also identified across institutional characteristics. Harm severity varied by type of healthcare institution (χ^2^ = 440.570, *p* < 0.001), with clinics showing the highest proportion of near-miss incidents (99.5%), while higher proportions of adverse/sentinel events were observed in general hospitals (24.5%) and tertiary hospitals (26.3%). Similarly, harm severity differed significantly by hospital bed capacity (χ^2^ = 502.845, *p* < 0.001), with the proportion of adverse/sentinel events increasing with larger bed capacity and reaching 27.7% in hospitals with ≥500 beds. With respect to the clinical context, harm severity differed significantly across medical departments (χ^2^ = 245.897, *p* < 0.001). The highest proportion of adverse/sentinel events was observed in emergency & critical care departments (40.6%), followed by diagnostic & support services (28.9%). In contrast, near-miss incidents were most common in pediatrics (83.1%) and psychiatry & mental health departments (86.5%). Differences in harm severity were also evident according to incident time (χ^2^ = 88.987, *p* < 0.001). Compared with the daytime period (07:00–14:59), higher proportions of adverse/sentinel events were observed during the evening (15:00–22:59; 24.4%) and nighttime (23:00–06:59; 28.9%) periods. Detailed distributions of medication-related incidents by harm severity according to patient, institutional, and incident characteristics are presented in Table 2.

### 3.3. Factors Associated with Adverse/Sentinel Events in Medication-Related Incidents

Factors associated with adverse/sentinel events in medication-related patient safety incidents were examined using multivariate logistic regression analysis, with near-miss incidents as the reference outcome. The results are presented in Table 3.

Regarding temporal trends, the likelihood of adverse/sentinel events increased significantly with calendar year (OR = 1.18, 95% CI 1.14–1.23). With respect to patient characteristics, female patients had significantly lower odds of adverse/sentinel events compared with male patients (OR = 0.86, 95% CI 0.77–0.95). Using patients aged 0–19 years as the reference group, those aged 20–59 years (OR = 0.63, 95% CI 0.44–0.90) and ≥60 years (OR = 0.85, 95% CI 0.75–0.95) showed significantly lower odds of adverse/sentinel events. These findings differ from the descriptive analysis, where older patients showed a higher proportion of adverse events, indicating that the observed crude differences may be confounded by institutional and clinical factors such as care setting and department.

In terms of type of healthcare institution, with general hospitals as the reference category, clinics were associated with markedly lower odds of adverse/sentinel events (OR = 0.08, 95% CI 0.01–0.70). Hospitals (OR = 0.63, 95% CI 0.43–0.92) and tertiary hospitals (OR = 0.83, 95% CI 0.73–0.94) also demonstrated significantly lower odds of adverse/sentinel events compared with general hospitals. Regarding hospital bed capacity, using institutions with no inpatient beds as the reference category, hospitals with <200 beds (OR = 2.47, 95% CI 0.32–19.00), 200–499 beds (OR = 4.12, 95% CI 0.54–31.16), and ≥500 beds (OR = 7.04, 95% CI 0.93–53.35) showed increased odds of adverse/sentinel events; however, these associations did not reach statistical significance. The wide confidence intervals observed for these estimates suggest limited precision, likely due to small sample sizes in certain categories. Therefore, these findings should be interpreted with caution. With respect to the medical department, using medical disciplines as the reference group, significantly higher odds of adverse/sentinel events were observed in diagnostic & support services (OR = 1.54, 95% CI 1.14–2.07) and emergency & critical care departments (OR = 2.14, 95% CI 1.73–2.64). In contrast, surgical disciplines (OR = 0.92, 95% CI 0.82–1.04), pediatrics (OR = 1.31, 95% CI 0.87–1.99), psychiatry & mental health (OR = 0.53, 95% CI 0.29–0.97), and others (OR = 0.73, 95% CI 0.53–1.00) showed lower or non-significant odds compared with medical disciplines. Regarding incident time, compared with incidents occurring during the daytime period (07:00–14:59), the likelihood of adverse/sentinel events was significantly higher during the evening (15:00–22:59) (OR = 1.44, 95% CI 1.29–1.62) and nighttime (23:00–06:59) periods (OR = 1.43, 95% CI 1.22–1.68). Variance inflation factors ranged from 1.21 to 3.84, indicating no significant multicollinearity between the type of healthcare institution and hospital bed capacity. These results support the stability and robustness of the regression model.

## 4. Discussion

### 4.1. Statement of Principal Findings

This nationwide study examined recent trends, harm severity, and factors associated with adverse or sentinel medication-related patient safety incidents in Korean hospitals using five years of national reporting data. Medication-related incidents showed a substantial increase over time and accounted for a growing proportion of all reported patient safety events. However, this observed increase should not be interpreted as a direct increase in the true incidence of medication-related harm, as it may partly reflect changes in reporting practices, including the introduction of mandatory reporting for serious incidents in 2021 and improvements in reporting culture and system awareness. In addition, the transition to a hybrid reporting system in 2021 may have influenced not only the volume of reported incidents but also the distribution of harm severity, which limits the direct comparability of trends across years and should be considered when interpreting longitudinal findings. Approximately one-fifth of medication-related incidents resulted in adverse/sentinel events, indicating a considerable burden of clinically meaningful harm. However, the observed increase in medication-related incidents should be interpreted in the context of the introduction of mandatory reporting for serious incidents in 2021, which may have contributed to increased reporting, in addition to potential increases in actual medication-related risks. Adverse outcomes were more likely to occur in large hospitals, emergency and critical care settings, and during evening or nighttime periods, highlighting the influence of patient characteristics, organizational context, and care environments on medication-related harm. In addition, differences between descriptive and multivariable findings observed in this study highlight the importance of interpreting adjusted results as independent associations, rather than direct reflections of crude distributions, particularly in the context of heterogeneous patient and institutional characteristics.

From a nursing practice perspective, these findings are particularly significant because nurses are the final checkpoint in the medication use process and are primarily responsible for medication administration and monitoring. However, it should be noted that the present dataset does not include direct information on nursing staffing, workload, or specific nursing practices. Therefore, these interpretations should be understood as contextual implications rather than direct evidence derived from the data. Strengthening nursing surveillance, clinical judgment, and safe medication administration systems is essential to reducing preventable medication-related harm.

### 4.2. Interpretation Within the Context of the Wider Literature

The higher likelihood of adverse/sentinel events observed in general and tertiary hospitals is consistent with previous studies demonstrating increased medication-related harm in settings that manage patients with greater clinical complexity, multiple comorbidities, and polypharmacy [3,4]. Large hospitals also use high-alert medications more frequently, including anticoagulants, antineoplastic agents, insulin, and critical care drugs, which are well known to be associated with severe harm when errors occur [19,20].

In these environments, nurses play a pivotal role in managing high-alert medications, conducting double-check procedures, verifying dosing accuracy, and continuously assessing patients for early signs of deterioration. The increased harm observed in larger institutions may therefore indicate the need for enhanced nurse staffing models, structured independent double-check systems, and standardized medication administration protocols, particularly for high-risk drugs.

Similarly, the elevated risk of adverse outcomes in emergency and critical care settings aligns with prior evidence. These environments are characterized by rapidly changing patient conditions, time pressure, frequent medication adjustments, and complex dosing regimens. Systematic reviews and observational studies have consistently shown that medication administration errors and harmful events occur more frequently in intensive care units and emergency departments than in general wards [7,21,22]. The use of continuous infusions, weight- and renal function-adjusted dosing, and concurrent administration of multiple high-risk drugs further increases the potential for harm in these settings [21].

In emergency and critical care units, nurses must integrate rapid clinical assessment with complex pharmacological knowledge while operating under significant cognitive and environmental load. This finding highlights the importance of advanced nursing competencies, simulation-based medication safety training, and the implementation of technologies such as smart infusion pumps and barcode medication administration (BCMA) systems to support safe practice.

The association between larger hospital bed capacity and increased harm severity underscores the role of organizational complexity and multidisciplinary care processes. While multidisciplinary care offers important benefits, inadequate coordination and communication breakdowns across prescribing, dispensing, preparation, and administration processes can increase the risk of medication errors [23]. From a systems perspective, complex work environments with multiple handoffs are more vulnerable to cascading failures, increasing the likelihood that errors reach patients and result in harm [9].

For nursing practice, this finding highlights the need for structured handoff communication tools, standardized medication reconciliation processes led by nursing staff, and interprofessional collaboration frameworks that clarify roles and accountability across the medication management pathway. Nurse managers and clinical leaders should actively monitor workflow complexity and foster a safety culture that encourages incident reporting and near-miss learning.

The finding that adverse/sentinel events were more likely during evening and nighttime periods is also consistent with prior literature. Reduced staffing levels, increased workload, fatigue, and limited access to immediate senior clinical support during off-hours have been associated with higher rates of medication errors and adverse patient outcomes, including medication-related harm [24,25].

These temporal patterns have direct implications for nursing workforce management. Ensuring adequate nurse-to-patient ratios during evening and night shifts, minimizing extended working hours, and implementing fatigue mitigation strategies are critical components of medication safety. Additionally, strengthening clinical supervision and decision-support access during off-hours may help mitigate the increased vulnerability to medication-related harm observed in this study.

These findings are consistent with studies using national incident reporting systems in other countries. For example, analyses of the UK National Reporting and Learning System (NRLS) and similar databases have demonstrated that medication-related harm is more frequently reported in high-acuity settings and large hospitals while also emphasizing the influence of reporting practices and system-level factors on observed trends [4]. Such comparisons support the generalizability of the present findings while reinforcing the need for cautious interpretation of reporting-based data.

### 4.3. Strengths and Limitations

This study has several notable strengths. The use of a large, nationwide patient safety reporting dataset enabled comprehensive analysis across diverse hospital types, clinical departments, and care environments. In addition, distinguishing near-miss incidents from adverse/sentinel events allowed for the identification of factors specifically associated with clinically meaningful harm. From a nursing perspective, the inclusion of diverse clinical settings strengthens the applicability of the findings to frontline medication administration and monitoring practices across hospital units.

However, several limitations should be acknowledged. Incident reporting systems are inherently subject to underreporting and reporting bias, and the true incidence of medication-related harm is likely underestimated. In addition, the exclusion of cases with missing incident time and clinical department may have introduced selection bias, as reporting completeness may vary across institutions and clinical settings. These excluded cases may differ systematically from the analyzed sample, as missing information may be associated with specific healthcare settings or institutional reporting practices. Because a formal comparison between included and excluded cases was not feasible within the constraints of the available secondary dataset, the magnitude of potential selection bias could not be quantified directly and should be considered when interpreting the findings. Furthermore, the absence of denominator data, such as total admissions or patient-days, limits the ability to estimate incidence rates and compare risk across institutions. In particular, variation in reporting culture and workload across nursing units may have influenced reporting frequency and severity classification. Sentinel events were relatively infrequent and therefore analyzed in combination with adverse events, which limited more detailed examination of the most severe outcomes. Finally, the cross-sectional design of the analysis precludes causal inference. In addition, variation in reporting practices across institutions and potential clustering of observations within hospitals may have influenced the results, as institutional-level effects were not explicitly accounted for in the analysis. Furthermore, the exclusion of a substantial number of cases due to missing data may have introduced selection bias, as excluded records may differ systematically from the analyzed sample. In addition, some regression estimates, particularly those related to hospital bed capacity, showed wide confidence intervals, indicating limited precision and potential instability of estimates due to small sample sizes in certain categories.

### 4.4. Implications for Policy, Practice, and Research

The findings suggest that medication-related harm is associated with multiple interacting factors, including patient characteristics, organizational context, and care environments. However, because this study is based on incident reporting data, these findings reflect associations rather than causal relationships. Medication safety interventions should therefore move beyond uniform approaches and instead focus on context-specific, targeted strategies. Large hospitals, emergency departments, intensive care units, and evening or nighttime shifts represent priority areas for enhanced medication safety interventions. These findings highlight the importance of system-level approaches involving multidisciplinary collaboration across healthcare professionals, rather than focusing solely on individual-level interventions.

For nursing practice, this implies the need for strengthened medication administration protocols, structured double-check systems for high-alert medications, and enhanced clinical surveillance in high-risk environments. These recommendations should be interpreted as system-level implications informed by observed patterns, rather than direct evidence on staffing levels or workload effects, which were not measured in the present study. Nurse managers may consider staffing adequacy and workload distribution as potential factors relevant to medication safety, although these aspects were not directly examined in the present study.

At the policy level, national patient safety strategies may benefit from incorporating risk stratification based on care setting and time of occurrence. In parallel, international accreditation survey processes have been associated with improvements in medication safety practices, reporting behaviors, and multidisciplinary coordination, suggesting that structured external evaluation mechanisms may reinforce system-level medication safety governance [26]. In practice, organizational support strategies, including appropriate staffing considerations, decision support, and medication safety programs tailored to high-risk environments, supported by structured evaluation and accreditation systems, may contribute to reducing preventable harm. Integrating nursing-sensitive indicators and medication safety competencies into national quality improvement frameworks may further support sustainable safety improvements. Future research should explore longitudinal associations, examine specific medication classes, and evaluate the effectiveness of targeted interventions using national reporting data.

## 5. Conclusions

This nationwide analysis provides robust evidence on recent trends and determinants of medication-related patient safety incidents in Korean hospitals. Reported medication-related incidents showed an increasing trend, and a substantial proportion resulted in clinically meaningful harm. However, these findings should be interpreted cautiously, as they are based on reported incidents and do not represent the true incidence of medication errors. These findings should also be interpreted with caution, as changes in reporting practices, including the introduction of mandatory reporting for severe incidents in 2021, may have influenced both the volume of reported cases and the distribution of harm severity, thereby limiting direct interpretation of temporal trends. Adverse outcomes were associated with organizational and contextual factors, suggesting the need for targeted, system-level medication safety strategies to reduce preventable harm in high-risk patients and care environments.

Given the multidisciplinary nature of medication management, system-level strategies should extend beyond nursing-focused interventions to include coordinated efforts among physicians, pharmacists, nurses, and healthcare organizations, supported by integrated safety systems and interprofessional collaboration. In particular, the significantly higher risk of adverse/sentinel events identified in emergency and critical care settings highlights the need for policy and practice interventions tailored to these high-risk environments, including enhanced clinical decision support, standardized protocols for high-alert medications, and consideration of appropriate staffing and supervision during high-intensity care periods. Aligning national patient safety policies with these high-risk contexts may help improve the effectiveness of medication safety strategies and contribute to reducing preventable harm at the system level.

## Figures and Tables

**Table 1:** Annual number and proportion of medication-related patient safety incidents reported to KOPS, 2020–2024.

Year	2020	2021	2022	2023	2024
Total reported incidents	13,919	13,146	14,820	20,273	22,118
Medication-related incidents	4325	4198	6412	10,089	11,257
Proportion of medication-related incidents (%)	31.10%	31.90%	43.30%	49.80%	50.90%

**Table 2:** Characteristics of medication-related incidents by harm severity.

Variable	Categories	Near-Miss	Adverse/Sentinel Event	Total	*χ* ^2^	*p*-Value
Year	2020	1225 (90.6)	127 (9.4)	1352 (100)	282.382	<0.001
	2021	1143 (69.9)	492 (30.1)	1635 (100)		
	2022	1419 (76)	449 (24)	1868 (100)		
	2023	1589 (73.9)	562 (26.1)	2151 (100)		
	2024	2108 (84.7)	381 (15.3)	2489 (100)		
Gender	Male	3616 (79.1)	954 (20.9)	4570 (100)	0.489	0.485
	female	3868 (78.5)	1057 (21.5)	4925 (100)		
Age	0–19	779 (84.3)	145 (15.7)	924 (100)	33.818	<0.001
	20–59	2390 (80.6)	575 (19.4)	2965 (100)		
	≥60	4315 (77)	1291 (23)	5606 (100)		
Type of healthcare institution	Clinic	1348 (99.5)	7 (0.5)	1355 (100)	440.57	<0.001
	Hospital	274 (88.4)	36 (11.6)	310 (100)		
	General hospital	3793 (75.5)	1229 (24.5)	5022 (100)		
	Tertiary hospital	2069 (73.7)	739 (26.3)	2808 (100)		
Bed capacity	<200 beds	332 (87.1)	49 (12.9)	381 (100)	502.845	<0.001
	200–499 beds	2006 (79.9)	504 (20.1)	2510 (100)		
	≥500 beds	3776 (72.3)	1450 (27.7)	5226 (100)		
	No inpatient beds	1370 (99.4)	8 (0.6)	1378 (100)		
Medical department	Medical disciplines	3134 (78)	883 (22)	4017 (100)	245.897	<0.001
	Surgical disciplines	2461 (78)	695 (22)	3156 (100)		
	Diagnostic & support services	177 (71.1)	72 (28.9)	249 (100)		
	Emergency & critical care	274 (59.4)	187 (40.6)	461 (100)		
	Pediatrics	526 (83.1)	107 (16.9)	633 (100)		
	Psychiatry & mental health	83 (86.5)	13 (13.5)	96 (100)		
	Others	829 (93.9)	54 (6.1)	883 (100)		
Incident time	Day (07:00–14:59)	4468 (82.1)	977 (17.9)	5445 (100)	88.987	<0.001
	Evening (15:00–22:59)	2304 (75.6)	745 (24.4)	3049 (100)		
	Night (23:00–06:59)	712 (71.1)	289 (28.9)	1001 (100)		
Total		7484 (78.8)	2011(21.2)	9495 (100)		

**Table 3:** Multivariate logistic regression analysis of factors associated with adverse/sentinel events in medication-related patient safety incidents.

Variable	Categories	OR	95% CI	
			LLCI	ULCI
Year		1.18	1.14	1.23
Gender (Ref: Male)	Female	0.86	0.77	0.95
Age	20–59	0.63	0.44	0.9
(Ref: 0–19)	≥60	0.85	0.75	0.95
Type of healthcare institution	Clinic	0.08	0.01	0.7
(Ref: General hospital)	Hospital	0.63	0.43	0.92
	Tertiary hospital	0.83	0.73	0.94
Bed capacity	<200 beds	2.47	0.32	19
(Ref: No inpatient beds)	200–499 beds	4.12	0.54	31.16
	≥500 beds	7.04	0.93	53.35
Medical department	Surgical disciplines	0.92	0.82	1.04
(Ref: Medical disciplines)	Diagnostic & support services	1.54	1.14	2.07
	Emergency & critical care	2.14	1.73	2.64
	Pediatrics	1.31	0.87	1.99
	Psychiatry & mental health	0.53	0.29	0.97
	Others	0.73	0.53	1
Incident time	Evening (15:00–22:59)	1.44	1.29	1.62
(Ref: Day (07:00–14:59))	Night (23:00–06:59)	1.43	1.22	1.68

## Data Availability

The data presented in this study are available from the Korea Patient Safety Reporting & Learning System (KOPS) at https://www.kops.or.kr (accessed on 20 August 2025). These data were derived from publicly available national patient safety incident reporting resources operated by the Ministry of Health and Welfare of the Republic of Korea.
